# Response of Development and Body Mass to Daily Temperature Fluctuations: a Study on *Tribolium castaneum*

**DOI:** 10.1007/s11692-016-9375-6

**Published:** 2016-02-24

**Authors:** P. Kramarz, D. Małek, K. Naumiec, K. Zając, S. M. Drobniak

**Affiliations:** Institute of Environmental Sciences, Jagiellonian University, Gronostajowa 7, 30-387 Krakow, Poland

**Keywords:** Insects, Development time, Body size, Thermal conditions, GEI

## Abstract

Differences in thermal regimes are of paramount importance in insect development. However, experiments that examine trait development under constant temperature conditions may yield less evolutionarily relevant results than those that take naturally occurring temperature fluctuations into account. We investigated the effect of different temperature regimes (constant 30 °C, constant 35 °C, fluctuating with a daily mean of 30 °C, or fluctuating with a daily mean of 35 °C) on sex-specific development time and body mass in *Tribolium castaneum*. Using a half-sib breeding design, we also examined whether there is any evidence for genotype-by-environment interactions (GEI) for the studied traits. In response to fluctuating temperature regimes, beetles demonstrated reaction norm patterns in which thermal fluctuations influenced traits negatively above the species’ thermal optimum but had little to no effect close to the thermal optimum. Estimated heritabilities of development time were in general low and non-significant. In case of body mass of pupae and adults, despite significant genetic variance, we did not find any GEI due to crossing of reaction norms, both between temperatures and between variability treatments. We have observed a weak tendency towards higher heritabilities of adult and pupa body mass in optimal fluctuating thermal conditions. Thus, we have not found any biasing effect of stable thermal conditions as compared to fluctuating temperatures on the breeding values of heritable body-size traits. Contrary to this we have observed a strong population-wide effect of thermal fluctuations, indicated by the significant temperature-fluctuations interaction in both adult and pupa mass.

**Electronic supplementary material:**

The online version of this article (doi:10.1007/s11692-016-9375-6) contains supplementary material, which is available to authorized users.

## Introduction

Temperature has profound effects on biological functions at all levels of organization (Hochachka and Somero 2002) and is undoubtedly one of the most important abiotic factors governing the lives of ectotherms such as insects. In nature, organisms are likely to experience daily temperature fluctuations (i.e., thermoperiods); however, most experiments in thermal biology are performed using constant temperatures. Consequently, results from such experiments may be less relevant evolutionarily and physiologically than results from experiments that take temperature fluctuations into account (see Colinet et al. 2015 for a recent review).

**Fig. 1:**
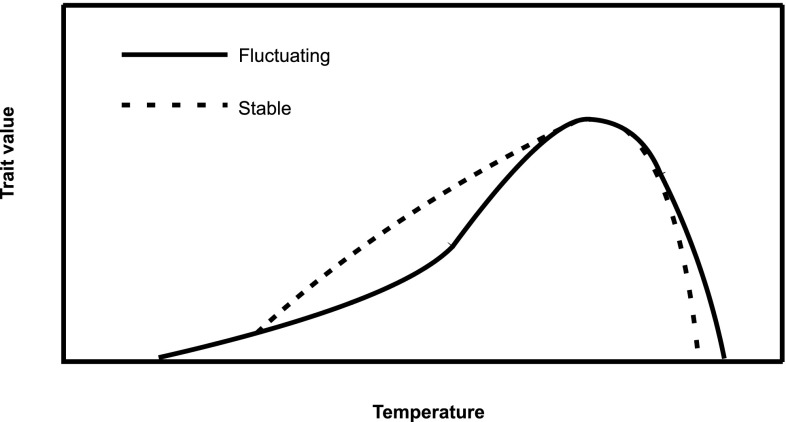
Theoretical influence of temperature fluctuations on trait values as expected by Jensen’s inequality. (Based on Kjærsgaard et al. 2013). *Solid line* Stable thermal conditions; *Dashed line* Fluctuating thermal conditions

The specific mechanism that governs the effects of thermal fluctuations is still under debate. Kjærsgaard et al. (2013) recently suggested that the effects of fluctuations vary due to vary due to their amplitude, average temperature and the shape of the function describing the thermal reaction norm. Such functions typically have three phases: (1) at low temperatures, there is an acceleration phase in which small increases in temperature are followed by nonlinearly large increases in a given trait value; (2) at intermediate temperatures, there is a linear phase in which changes in temperature result in proportional changes in a trait; and (3) at high temperatures, there is a rapid deceleration phase in which increases in temperature are increasingly detrimental for the trait of interest (Schoolfield et al. 1981). To characterize the effects of variance in temperature on this function, a mathematical phenomenon called Jensen’s inequality (Ruel and Ayres 1999) must be taken into account. According to Jensen’s inequality, variance in thermal conditions depresses the response variable in the deceleration phase of the function, elevates it in the acceleration phase, and leaves it unchanged in the linear phase (see Fig. 1 for illustration). Additionally, a range amplitude of thermal fluctuations has to be taken into account, as results may differ when fluctuations encompass extremely stressful temperatures, thus changing the effect. For example, high temperature during may limit the time of activities such as searching for sexual partners or foraging and thus influence overall fitness. The latter may also prolong fasting period and, despite energy savings during cooler period, lead to higher energetic requirements in fluctuating vs. stable thermal conditions (see Colinet et al. 2015 for review).

In thermal biology, body size is often the trait of interest as it is an important fitness-related trait that affects all aspects of an individual’s physiology (e.g., Roff 1992; Stearns 1992). It also exhibits substantial plasticity in response to variation in thermal conditions during an individual’s development (Davidowitz and Nijhout 2004; Chown and Gaston 2010). Final achieved body size is strongly dependent on the total time of development (and consequently the duration of individual developmental stages), because it is the product of the amount of time available for growth and the rate at which mass is accumulated during that period (Davidowitz and Nijhout 2004). This point has been widely discussed (see for example: Atkinson 1994) as it underlies one of the most important life-history trade-offs between maturation time and size at maturity (Roff 1992; Stearns 1992). While the influence of mean temperature on body size is quite well-studied (Atkinson 1994) there are still many unknowns with regard to the response of both development time and body size to fluctuating temperature. The main reason for this is the fact that despite an increasing number of studies on thermal fluctuations relatively few of them deal with body size (but see: Brakefield and Kesbeke 1997; Fischer et al. 2011; Kjærsgaard et al. 2013).

Plastic responses of certain traits to changing temperatures are expected to be of importance to their evolutionary dynamics if plasticity is coupled with genetic trade-offs between two contrasting environments (e.g., temperatures). If genotypes vary in their response to environmental variation, such pattern is referred to as a genotype-by-environment interaction (GEIs; Falconer 1981). GEIs can be expressed as a decrease in the genetic correlation between the breeding values of a trait in different environments (Falconer 1952; Via 1987) and can also be seen as the measure of the ability of that trait to evolve independently in those environments (Via and Lande 1985). GEIs that involve temperature and its variability are nowadays a subject of intensive studies (see for example: Brakefield and Kesbeke 1997; Ketola et al. 2012; Bozinovic et al. 2011) due to their importance to understanding the genetics of populations occupying changing environments (Gienapp and Brommer 2014).

Here we aim to: (1) explore the effects of temperature and its variability on insect development time and body mass during different developmental stages, and (2) test for the presence of GEIs generated by temperature and its variability in the mentioned traits. To achieve this we used a nested half-sib/full-sib design (Lynch and Walsh 1998) to examine body mass and development time in different developmental stages of the red flour beetle (*Tribolium castaneum* Herbst, 1797) exposed to “normal”, and “elevated” temperatures, crossed with two variability treatments: fluctuating and constant thermal regime.

Despite the fact that *T. castaneum* is originally a tropical species (Sokoloff 1975), nowadays it can be found across all climate zones in flour mills and other grain-processing facilities. Such habitats are characterized by daily and seasonal temperature fluctuations (Campbell et al. 2010). To our knowledge, the only data on the influence of thermal variability on the development and body mass of *T. castaneum* come from our previous study (which for technical reasons used a different strain of this species), in which we found that thermal fluctuations hastened development and increased body mass compared to constant conditions in treatments with mean temperatures below the thermal optimum (25 °C; Małek et al. 2015).

## Materials and Methods

### Experimental Animals

The beetles in this study was kindly provided by Barbara Milutinović (see CR-01 in Milutinović et al. 2013). The strain is kept outbred at a constant temperature of 30 °C (the “normal” temperature in this study) in constant darkness and fed ad libitum on a medium composed of organic wheat flour and yeast (9:1 ratio). *Tribolium castaneum* beetles do not need additional water sources as they absorb humidity from the substrate. (Sokoloff 1975). The beetles are kept in plastic boxes with lids that contain ventilation holes made from steel mesh; the humidity in culture is 70 % RH. Experimental animals were reared in the laboratory conditions for approximately 35 generations and kept outbred. It is worth mentioning that genetic diversity is often reduced in laboratory conditions (Briscoe et al. 1992). This is an important aspect that may have played a role in our ability to detect the presence of GEI.

### Experimental Design

**Fig. 2:**
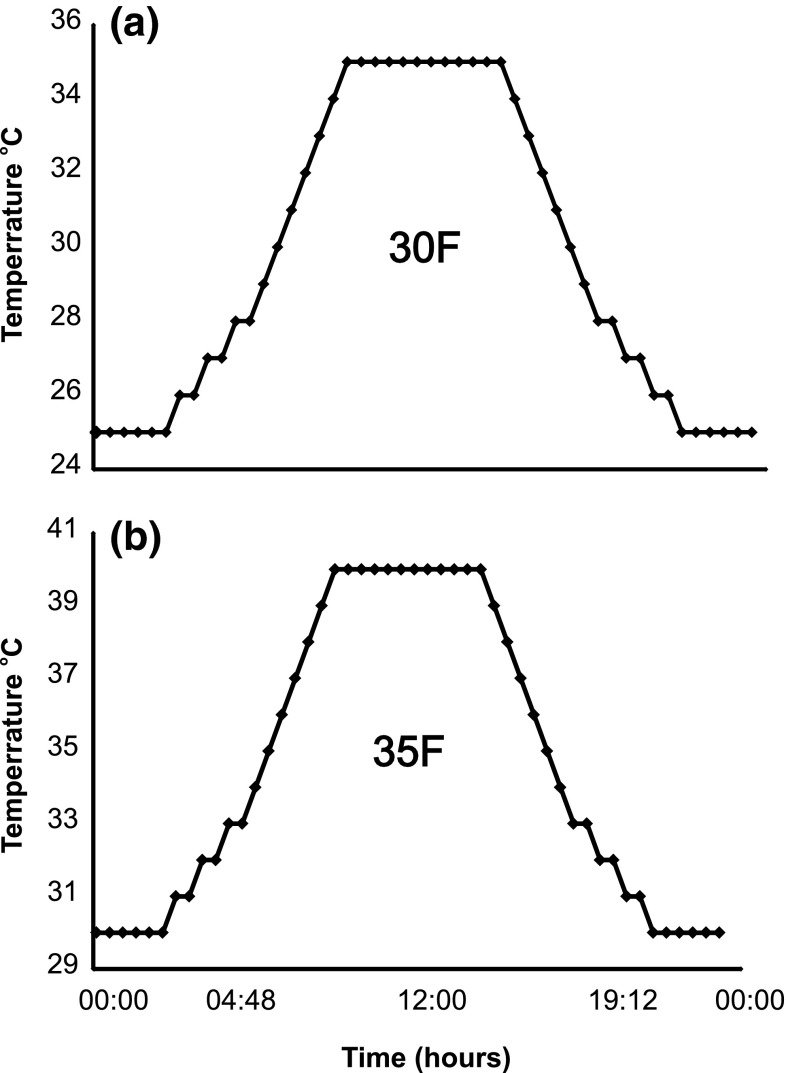
Scheme of applied thermal fluctuations. **a** Mean temperature of 30 °C; **b** mean temperature of 35 °C

We exposed beetle eggs to one of four temperature regimes: normal constant (30 °C, hereafter abbreviated as 30S), elevated constant (35 °C—35S), normal fluctuating (fluctuating around a daily mean of 30 °C—30F), or elevated fluctuating (fluctuating around a daily mean of 35 °C—35F). In all climatic chambers the humidity was 70 % RH. In the two treatments in which temperatures fluctuated, fluctuations took place over the course of the day and mimicked real diurnal temperature patterns (Fig. 2). Temperatures increased in the morning, reached a plateau near noon, cooled in the evening, and reached a stable night-time low. Other conditions were as in main culture.

During the experiment, 35 randomly selected males (sires) were each transferred to a smaller individual box and mated to four randomly selected females (dams). Females were then isolated and allowed to lay eggs. Every 24 h, each female’s newly laid eggs were transferred into one of four different temperature regimes. The females and their offspring were provided unlimited access to food. Because of low female fecundity and high offspring mortality, our sample for quantitative genetic analysis was limited to 2.957 offspring produced by 53 dams, which were distributed across 19 sires (mean of 2.8 ± 0.6 dams per sire; range 1–4). The treatment-specific sample sizes (N) were as follows: 30S: N = 790, 30F: N = 769, 35S: N = 756, 35F: N = 642. For both sexes of half-sib progeny across all treatments, we measured the development time of all stages (time to pupation, length of pupal stage, and time to adult emergence) and pupal and adult body mass.

### Statistical Methods

Data was analyzed with general linear mixed models fitted in ASReml-R (Gilmour et al. 2009). Each model included (as fixed effects) sex, fluctuating versus stable treatment, two established temperature treatments, and their interaction. Significance of fixed terms was tested using a conditional Wald tests and non-significant interactions were eliminated. In order to simplify quantitative genetic analyzes we have modified this set of fixed effects in models aimed at estimating treatment-specific genetic (co)variances: in these models the setup employing thermal treatment, variability treatment and their interaction was replaced by a 4-categories fixed variable representing all combinations of the two experimental treatments. Mathematically it is equivalent with the interaction representation, but allows for straightforward definition of the 4-by-4 G-matrix in ASReml-R.

All models included dam and sire as random effects. Sire variance (*V*_*sire*_) was used to estimate the heritability (computed as *h*^*2*^ = 4 *V*_*sire*_/(*V*_*sire*_ + *V*_*dam*_ + *V*_*residual*_); Lynch and Walsh 1998). Standard errors of heritabilities were estimated using the delta method (Lynch and Walsh 1998). Random effects and (co)variance structures were tested by comparing respective models with and without the focal random term via a likelihood-ratio test with an appropriate number of degrees of freedom (equal to the difference in the number of (co)variance parameters between the complex and simplified model). E.g., significance of genetic variance components was tested by eliminating the sire term from the model and comparing its likelihood to the likelihood of the full model. In tests involving variances we have used a correction that takes into account the bounded character of variance parameters (variances are always non-negative; Self and Liang 1987). If the test statistic *LRT* = 2log(*likelihood*_1_/*likelihhod*_*2*_) has the asymptotic distribution χ_r2−r1_^2^, where *r*_*1*_ and *r*_*2*_ are parameter numbers of respective models, than for variances the appropriate *P* value for this statistic is 0.5(1−Pr(χ_r2−r1_^2^ ≤ *lrt*), where *lrt* is the observed value of *LRT* (Self and Liang 1987).

To test for the presence of genotype-by-environment interactions (GEI) between the two temperatures we have fitted 4-variate (see above) models considering traits expressed in all four combinations of treatments as four response variables. GEI may have two components, not mutually exclusive (Charmantier et al. 2015; Hoffmann and Merilä 1999): (i) heritabilities of traits may differ significantly between two environments; (ii) reaction norms between two environments may cross, resulting in significantly less than unity cross-environment genetic correlations. Thus, we have estimated trait heritabilities in both thermal environments and tested the sign and magnitude of cross-temperature genetic correlations.

The tests of varying kinds of GEI were performed via a series of likelihood-ratio tests using models of increasing complexity. The following models were fitted; we refer to their code-names in the results section:

- No heterogeneity in sire/dam/residual variances, constrained to unity: id(G), id(M), id(R);
- Heterogenous dam variances, covariances fixed at zero; sire and residual effects—as above: id(G), idh(M), id(R);
- Heterogenous residual variances, covariances fixed at zero; sire and dam effect—as above: id(G), id(M), idh(R);
- Heterogenous sire and residual variances, covariances fixed at zero; dam effects homogenous: idh(G), id(M), idh(R);
- Heterogenous residual variance, covariances fixed at zero; unconstrained sire effects (heterogenous variances and unconstrained covariances); dam variances homogenous: us(G), id(M), idh(R);
- Heterogenous residual variances, covariances fixed at zero; unconstrained sire effects (heterogenous variances but correlations fixed at unity); dam variances homogenous: corh(G), id(M), idh(R).

Due to computational limitations of our data set (most probably not large enough number of sires) the models fitting more complex dam effect structures failed to converge—thus we have limited our most complex dam models to the case of unconstrained dam variances without estimating dam covariances. Similar problem disallowed us to fit complex heterogenous covariance structures for the sire effect in which some correlations are constrained and some are unconstrained. Thus only fully constrained/unconstrained models were analyzed.

Each individual was measured only in one of the environments and hence cross-environmental residual covariance was fixed at zero, as it is not identifiable. Residual variances were fitted separately in both environments (i.e., allowing for different residual variances in different temperatures or stable/fluctuating conditions) to avoid bias in heritability estimates resulting from ignoring differing residual variances.

For pupal and adult body masses and for mass reduction during pupation, additional linear mixed models were used to account for effects of larval stage length, pupal stage length, and total development time respectively (see the supplementary materials).

## Results

### Fixed Effects

**Fig. 3:**
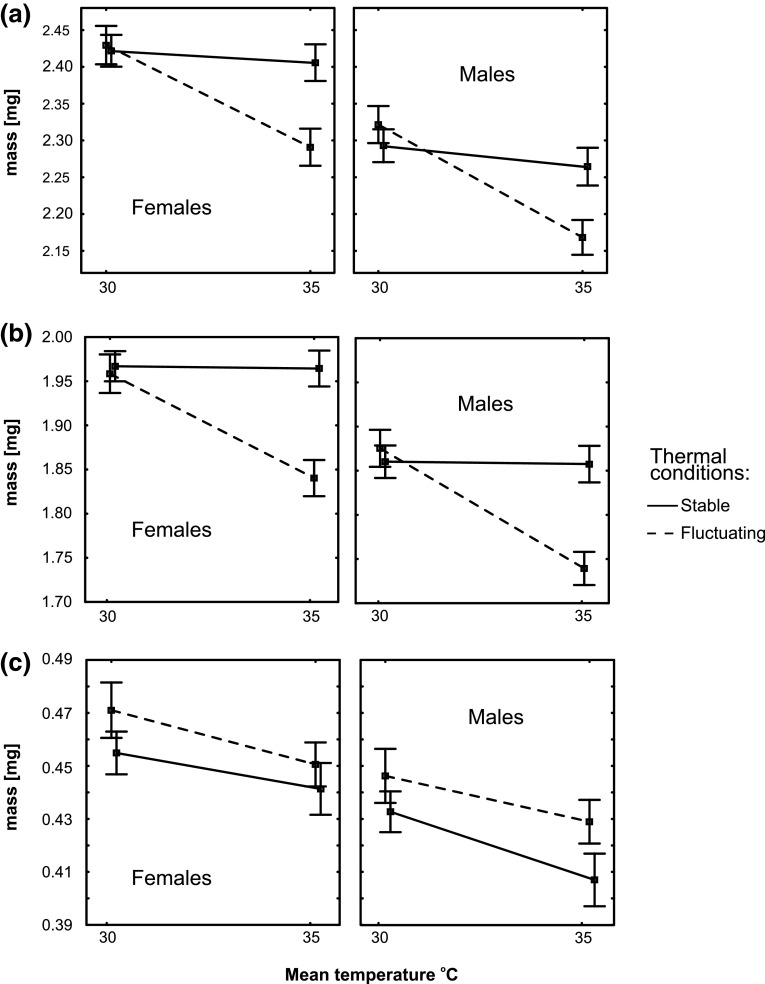
Body mass values in different temperatures and thermal fluctuation regimes in different life stages of *Tribolium castaneum* in both sexes. **a** Pupal body mass; **b** adult body mass; **c** mass reduction during pupation. *Bars* indicate 95 % confidence intervals

Body mass was significantly affected by temperature and thermal fluctuations (Table 1, Fig. 3). Sexes exhibited significant sexual dimorphism, with males being significantly smaller both at the pupa and adult stages (Fig. 3). Temperature and variability treatment formed a significant interaction (Table 1, Fig. 3), expect for mass reduction during pupation, where fluctuations and temperature influenced the response variable independently. In case of body mass of adults and pupae the reduction of mass in 35° compared to 30 °C was significant only in fluctuating thermal conditions (Table 1, Fig. 3).

**Table 1:** Results of general linear mixed models for pupal (a), adult (b) body mass and body mass reduction during pupation (c) of *Tribolium castaneum*

Factor	Numerator *df*	Denominator *df*	Adjusted F	*P*
a. Pupal mass				
	1	18	8127	<0.001
Temperature	1	2922	135.6	<0.001
Thermal fluctuations	1	2919	39.47	<0.001
Sex	1	2912	278.7	<0.001
Temperature × thermal fluctuations	1	2917	77.37	<0.001
b. Adult mass				
	1	18.2	7852	<0.001
Temperature	1	2924	113.5	<0.001
Thermal fluctuations	1	2920	95.52	<0.001
Sex	1	2913	260	<0.001
Temperature × thermal fluctuations	1	2918	110.6	<0.001
c. Mass reduction during pupation				
	1	16.2	4986	<0.001
Temperature	1	2935.7	45.94	<0.001
Thermal fluctuations	1	2929.6	18.50	<0.001
Sex	1	2920	67.42	<0.001

Model included dam and sire identities as random effects, unconstrained covariances among experimental treatments, and heterogeneous random effect variances

**Fig. 4:**
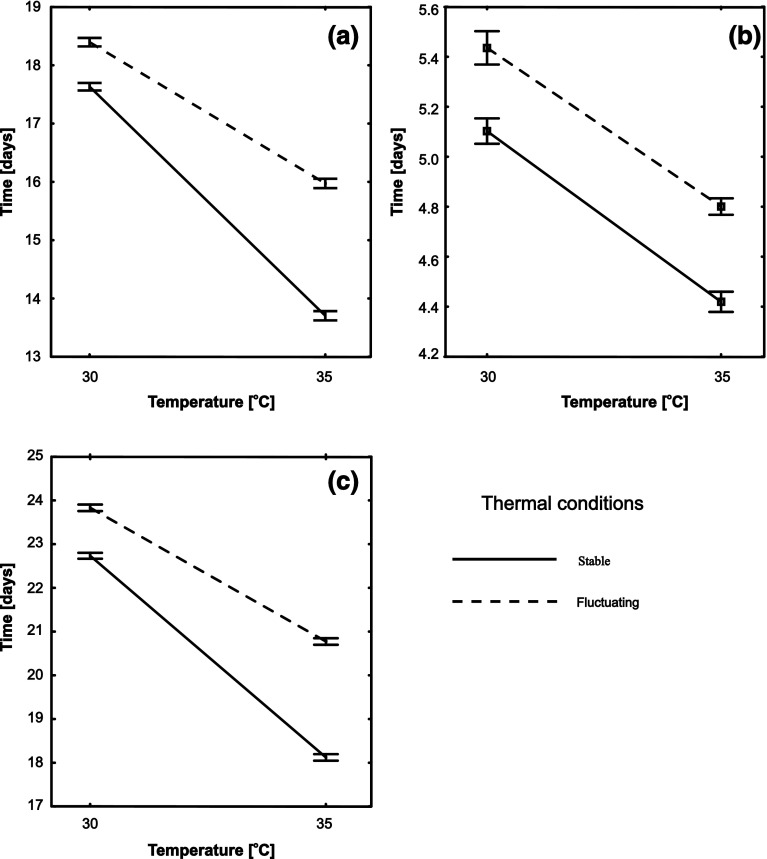
Reaction norms of development times in different temperature and thermal fluctuation regimes in different life stages of *Tribolium castaneum*. **a** Time needed by a larva to achieve pupation; **b** length of the pupation process; **c** total development time. *Bars* indicate 95 % confidence intervals

Development time was affected by both temperature and its fluctuations: individuals developing in 35 °C grew significantly faster (Table 2, Fig. 4) and this difference was more pronounced in stable conditions (except for pupation time, where temperature and fluctuations did not generate a significant interaction; Table 2, Fig. 4). Sexes did not exhibit dimorphism in time of development (Table 2, Fig. 4).

**Table 2:** Results of general linear mixed models for development time of *Tribolium castaneum*; (a) time to pupation; (b) time of pupation; (c) total development time

Factor	Numerator *df*	Denominator *df*	Adjusted F	*P*
a. Time to pupation				
	1	35.2	61,620	<0.0001
Temperature	1	2906	8134	<0.0001
Thermal fluctuations	1	2924	1798	<0.0001
Sex	1	2919	0.386	0.534
Temperature × thermal fluctuations	1	2913	479.8	<0.0001
b. Time of pupation				
	1	15.2	75,010	<0.0001
Temperature	1	2950	1148	<0.0001
Thermal fluctuations	1	2934	430	<0.0001
Sex	1	2934	1.799	0.179
Temperature × thermal fluctuations	1	2938	48.57	<0.0001
c. Total development time				
	1	16.5	81,460	<0.0001
Temperature	1	2925	11,940	<0.0001
Thermal fluctuations	1	2920	2898	<0.0001
Sex	1	2908	0.016	0.900
Temperature × thermal fluctuations	1	2915	648	<0.0001

Model included dam and sire identities as random effects, unconstrained covariances among experimental treatments, and heterogeneous random effect variances

### Heritabilities

Duration of development did not exhibit any significant genetic effects: in all three traits (time to pupation, time of pupation and total development time) models excluding sire effect did not have higher likelihoods compared to models including this effect (Table 3) and achieved low heritabilities ranging from 8 to 12 %. Body mass traits were heritable—in both pupa mass and adult mass, models including the genetic (sire) effect had higher; Table 3). Heritabilities of mass traits were moderate, ranging from 44 to 51 % (Table 3).

**Table 3:** Estimates of dam, sire (genetic) and residual variances in all analyzed traits in univariate models accounting for relevant fixed effects

Trait	Sire var.	Sire P	Dam var.	Dam P	Residual	h^2^	h^2^ SE	m^2^	m^2^ SE
Pupa mass	0.102	0.043	0.187	<0.001	0.649	0.44	0.26	0.09	0.11
Adult mass	0.118	0.014	0.154	<0.001	0.655	0.51	0.26	0.04	0.1
Pupa development time	<0.001	1.000	0.041	<0.001	0.204	<0.001	Boundary	0.17	0.04
Adult development time	0.004	0.132	0.002	0.5	0.634	0.02	0.02	−0.003	0.008
Total development time	0.004	0.427	0.025	<0.001	0.151	0.09	0.13	0.12	0.06
Mass difference (adult—pupa mass)	0.033	0.195	0.093	<0.001	0.854	0.14	0.12	0.06	0.05

We provide dam and sire variances with their *P* values (based on *df* = 1 likelihood-ratio test) and residual variances. Additionally, traits’ heritabilities (h^2^ = 4V_sire_/(V_sire_ + V_dam_ + V_residual_), with their SEs) and proportions of variance explained by maternal effects (m^2^ = (V_dam_-V_sire_)/(V_sire_ + V_dam_ + V_residual_), with their SEs) are provided

### Genotype-by-Environment Interactions (GEI)

Testing for GEI we have considered only traits where inclusion of the sire (genetic) effect resulted in a significant increase of model likelihood (i.e., pupa mass and adult mass). None of the estimated cross-treatment genetic correlations was significantly different from unity: although models constraining only some of the correlations did not converge, the model that constrained all 6 correlations to unity was the preferred one (Table 4, *P* = 0.48 for pupa mass, *P* = 0.49 for adult mass; Table 5). Model comparisons indicated that, having constrained genetic correlations to one, the model with heterogenous varying genetic variances was the preferred model (Table 4, *P* < 0.001 for both pupa and adult mass). The resulting covariance matrices indicated that heritabilities in 35° and fluctuating temperatures tended to be lower, both in pupa and adult mass, compared to heritabilities in the remaining treatment combinations (Table 5).

**Table 4:** Sequential tests of (co)variance structures with increasing complexity (see the “Methods” Sect)

Model 1			Model 2			ΔlogL	*df*	*P*
Sire model	Dam model	R model	Sire model	Dam model	R model			
Pupa mass								
id(G)	id(M)	idh(RR)	id(G)	id(M)	id(RR)	12.62	3	<0.001
id(G)	idh(M)	idh(RR)	id(G)	id(M)	idh(RR)	2.03	3	0.12
idh(G)	id(M)	idh(RR)	id(G)	id(M)	idh(RR)	1.73	3	0.16
corh(G)	id(M)	idh(RR)	id(G)	id(M)	idh(RR)	25.54	3	<0.001
us(G)	id(M)	idh(RR)	corh(G)	id(M)	idh(RR)	0.26	1	0.48
Adult mass								
id(G)	id(M)	idh(RR)	id(G)	id(M)	id(RR)	16.52	3	<0.001
id(G)	idh(M)	idh(RR)	id(G)	id(M)	idh(RR)	0.61	3	0.37
idh(G)	id(M)	idh(RR)	id(G)	id(M)	idh(RR)	1.61	3	0.12
corh(G)	id(M)	idh(RR)	id(G)	id(M)	idh(RR)	26.9	6	<0.001
us(G)	id(M)	idh(RR)	corh(G)	id(M)	idh(RR)	0.12	5	0.49

Models were fitted for traits were significant genetic variance was detected. We provide the structures of the simple (i.e., constrained, 1) and complex (2) model, for each comparison we provide difference in logged models’ likelihoods (complex model minus simple model), degrees of freedom (*df*) equal to the number of parameters differentiating the two models and *P* values assuming that 2Δlog(L) is distributed as *χ* ^*2*^ with appropriate *df*

**Table 5:** Estimates of G-matrices (approximated by the sire effect) for pupa and adult body mass

	30 F	35 F	30 S	35 S
*a. Pupa mass*				
30 F	0.203	0.203	0.142	0.127
	0.78 (0.30)			
35 F	0.99 (0.34)	0.079	0.222	0.112
		0.39 (0.24)		
30 S	1.00 (0.24)	0.99 (0.35)	0.142	0.239
			0.74 (0.31)	
35 S	0.99 (0.24)	0.98 (0.40)	0.99 (0.23)	0.221
				0.80 (0.35)
*b. Adult mass*				
30 F	0.231	0.231	0.134	0.124
	0.86 (0.31)			
35 F	0.99 (0.32)	0.077	0.185	0.1
		0.39 (0.24)		
30 S	0.99 (0.18)	0.99 (0.33)	0.134	0.148
			0.72 (0.29)	
35 S	0.99 (0.22)	0.99 (0.39)	0.99 (0.20)	0.184
				0.72 (0.30)

Treatments are coded as 30/35 °C and fluctuating (F)/stable (S) conditions. Diagonal elements present sire variances (upper values) and heritabilities with standard errors (lower values + (SE)). Above-diagonal elements present covariance estimates from an unconstrained model (us(G), see “Methods”), below-diagonal elements represent cross-treatment genetic correlations (r = COV_1,2_/sqrt(V_1_V_2_))

Heterogenous dam variances were not supported (*P* = 12 and *P* = 37 for pupa mass and adult mass, respectively, Table 4). Residual variance turned out to be heterogenous between the experimental treatments (*P* < 0.001, Table 4).

**Fig. 5:**
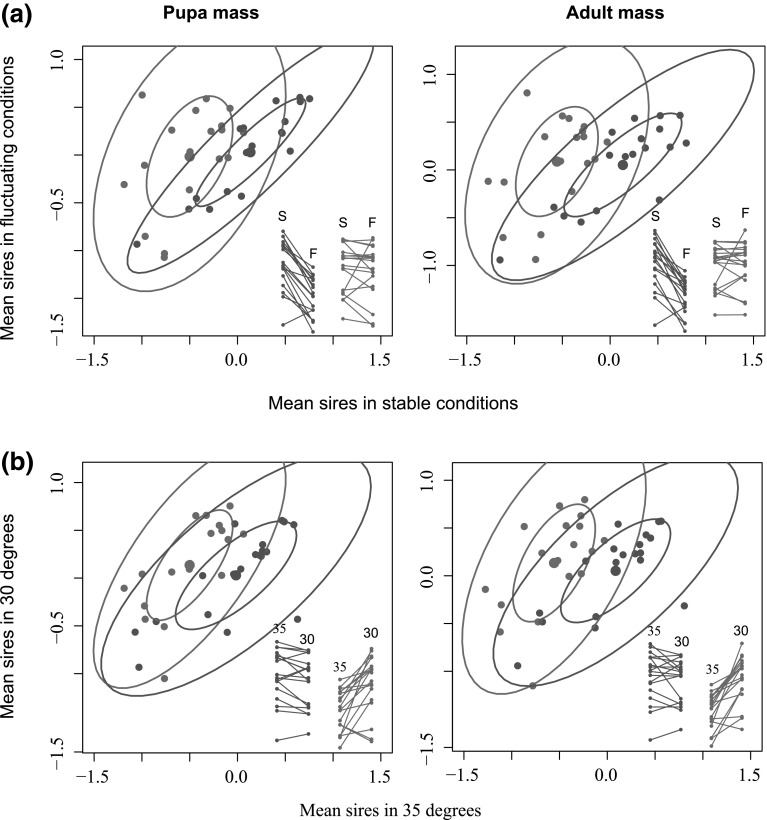
Cross-treatment average-sire phenotypic correlations between thermal variability treatments (**a**) and temperatures (**b**) for pupa mass and adult mass. **a** *red* variable thermal conditions; *blue* stable thermal conditions; **b** *red* 35 °C; *blue* 30 °C. Each point represents one sire. Inlets present reaction-norms’ plots, where *lines* connect same sires breeding in opposite treatment groups. *Colours* of inlet plots are analogous to correlational plots (Color figure online)

In all attempts to estimate cross-treatment genetic correlations the parameters were effectively fixed by ASReml-R at the boundary of parameter space (i.e., unity). It is difficult to determine whether it results from genuine lack of any crossing of reaction norms, or is due to lack of power. For illustration we have calculated estimates of phenotypic correlations for measurements averaged within sires. For adult mass, the cross-treatment correlation of averaged sires between variability treatments (with its 95 % confidence interval) was 0.43 (95 %CI 0; 0.75) in 35°, and 0.81 (95 %CI 0.56; 0.92) in 30° (Fig. 5a). Analogous correlation between temperature treatments was 0.57 (95 %CI 0.15; 0.82) for fluctuating conditions, and 0.72 (95 %CI 0.39; 0.88) for stable conditions (Fig. 5b). For pupa mass, the cross-treatment correlation of averaged sires between variability treatments was 0.46 (95 %CI 0.02; 0.76) in 35° and 0.90 (95 %CI 0.75; 0.96) in 30° (Fig. 5a). Analogous correlation between temperature treatments was 0.69 (95 %CI 0.34; 0.87) for fluctuating conditions, and 0.73 (95 %CI 0.42; 0.89) for stable conditions (Fig. 5b).

In general presented estimates are associated with extensive standard errors, which disallowed the calculation of more complex genetic parameters (such as variation in reaction norms slopes and elevations; Ketola et al. 2013): all of these parameters were approximated by the delta method as >1.0 and standard errors varying between 1.0 and 1.2. See “Discussion” for a more detailed account of the statistical power achieved in our quantitative genetic analyses.

## Discussion

We found that development time and body mass in *T. castaneum* were influenced in all developmental stages by both mean temperature and temperature variability, although the effects of the latter were significantly modulated by the former. At 30 °C, the “normal” (optimal) temperature (for both adults and larvae; see for example larval mortality in: Bucher 2009), fluctuations had either no effect or a slightly negative effect on trait values. However, at 35 °C, the “elevated” temperature, fluctuations significantly decreased body mass and extended development times. These data indicate that thermal fluctuations can significantly alter the outcome of experiments.

Jensen’s inequality predicts that temperature variability will have different effects on a reaction norm depending on the considered thermal interval of the reaction norm function; specifically, variance can enhance the response variable in the acceleration phase, depress it in the deceleration phase, or leave it unchanged in the linear phase (Ruel and Ayres 1999; Colinet et al. 2015). In the current study, we found that fluctuations caused little to no change in body mass at the optimal temperature, but lowered it at elevated temperature. For development time the situation was slightly different: fluctuations lengthened development time at both 35 and 30 °C, but to a visibly smaller degree at the lower temperature. When we compare the data from this study with those from our previous study of *T. castaneum* that examined temperatures below the thermal optimum (Małek et al. 2015, which for technical reasons used a different strain of *T. castaneum*), we find results that are consistent with this theory. In our previous study, thermal fluctuations hastened development and increased body mass compared to constant conditions at temperatures below the thermal optimum (25 °C in Małek et al. 2015) and, at optimal conditions, had effects similar to those presented here. We suggest that the reason for the incongruences observed between development time and body mass may be that the optimal temperature for the former is in fact different than that for the latter. The well-known but still poorly understood trend known as the Temperature-Size Rule may support this hypothesis. The Temperature-Size Rule states that individuals raised at low temperatures generally grow more slowly but finally become larger than those raised at higher temperature; such pattern has been reported in nearly 80 % of known ectothermic organisms in diverse taxa (Atkinson 1994). It is worth mentioning that in 35F treatment the animals spent about 5 h at 40 °C. Such temperature may be higher than the upper thermal threshold (CT_max_) of this species and the experimental animals may have experienced heat injuries during that period. Developmental time may therefore be delayed (increased) because of the necessary repair (and associated physiological cost) of the accumulated injuries when the temperature returns to more favorable conditions (Colinet et al. 2015).

Evolutionarily, the observed patterns would be of much greater interest if observed phenotypic trends were associated with underlying genetic trade-offs. From the point of view of quantitative genetics the studied traits were not equally heritable (approximate *t* test for extreme values in Table 1: *P* = 0.04). Estimated heritabilities of development time were in general low and non-significant, which is consistent with our previous study (Malek et al. 2015) but in contrast to some other studies (see for example Davidowitz et al. 2012; Prokkola et al. 2013; Rantala and Roff 2006). It is possible that combinations of employed experimental treatments disrupted genetic control over this trait, inflating the environmental/residual fractions of phenotypic variance. The estimated heritabilities of body size were also in line with the published estimates (approx. 0.5; for review see Nijhout and German 2012). Despite substantial genetic variance in the body mass of pupae and adults, we did not find any GEI due to crossing of reaction norms, both between temperatures and between variability treatments. However, we have observed a weak tendency towards lower heritabilitiess of adult and pupa body mass in non-optimal (35 degrees Celsius) fluctuating thermal conditions. Our stock population for many generation was exposed to stable thermal conditions, thus we can assume that any fluctuation in combination with non-optimal temperature can be stressful for it leading to downward changes in heritabilies (e.g., Hoffman and Merilä 1999).

Lack of evidence for significant crossing of reaction norms in our data should not disprove this possibility entirely—it likely results from power limitations of our dataset. We have obtained data coming from 19 sires, which is in general less than in most published studies using similar breeding designs. This fact is also likely causing inflated errors around most estimates and convergence problems of models attempting to estimate too many parameters at once. To provide a rough approximation of patterns that may be masked by low power we have also analyzed phenotypic values averaged across sires looking at their correlations between experimental treatments. In general, cross-temperature correlations were similar for both variability treatments in both heritable mass traits. However, when looking at cross-variability correlations they tended to be stronger in lower (i.e., optimal) temperatures. Thus, it seems that genetic integration of developmental traits such as body mass may be disrupted in non-optimal conditions. The determination of the degree and genetic basis of this disruption is however beyond the statistical power achieved in our study and requires further research.

Taken together with the results of Małek et al. (2015), current conclusions are a valuable contribution to the thermal biology literature because they add to a pool of studies considering the effects of temperature fluctuations both above and below a species’ known thermal optimum. Despite being a long recognized fitness modulator (see for example Ratte 1985), studies presenting similar patterns are still limited, especially in relation to traits studied here (but see Brakefield and Kesbeke 1997; Fischer et al. 2011; Kjærsgaard et al. 2013). At the same time, many studies of constant versus fluctuating temperatures focus on a single temperature, which often leads to contradictory conclusions among studies that investigate only low or only high temperatures. For example, body size in *Scatophaga stercoraria* was smaller under fluctuating than under constant temperature conditions (Kjærsgaard et al. 2013), but fluctuations had a positive influence on development time in *Lycaena tityrus* (Fischer et al. 2011). These results appear to be contradictory but this could be because we lack information about these species’ respective thermal optima. It is in agreement with a review by Lawson et al. 2015 which states that (for population growth) responses to changes in environmental variance are diverse and that increasing environmental variance can have a range of positive, neutral and negative effects, depending on the curvature of the trait. To resolve these inconsistencies in the future we suggest using both fluctuating and stable thermal regimes in studies in which the target species’ thermal optimum is known. In such studies the incorporation of temperature fluctuations into the experimental design can provide important insights into how that organism behaves under more realistic conditions, and accounting for stable temperatures may provide a valuable comparative evidence as such designs are still common in the field.

Apart from confirming theoretical expectations, our results have also some wider implications. For both development time and body mass, animal performance was worse in the fluctuating environment at the elevated temperature than in the constant treatment with the same mean. Ketola et al. (2012) found a similar relationship: lower egg-to-adult viability was observed in *Drosophila melanogaster* under cycling, rather than constant, 30 °C conditions. These findings are especially striking in the context of climate change. Many tropical species already live in environments with mean temperatures relatively close to their respective critical thermal maxima (CT_max_, see: Deutsch et al. 2008). Our results may suggest that predictions based only on constant temperatures may underestimate the effect of stressful elevated temperatures on those species. In a recent review by Lawson et al. (2015) authors proposed three main effects of environmental variance to be considered in relation to climate change: (1) the separate effects of changes in environmental means and variances may poorly approximate their combined effect; species might be able to deal with changes in either the mean or the variance of the environment, but be overwhelmed by simultaneous changes in both; (2) in the absence of local adaptation, populations in different locations may still respond differently to environmental change; (3) even if the magnitude of environmental variance remains constant, accounting for its effects may nonetheless be critical to predict population responses to changes in the mean environment. The authors also stressed that most existing population dynamical models either omit temporal environmental variation entirely or assume linear or quadratic population growth responses, what would cause the effects of environmental variance to be identical in all mean environments (Lawson et al. 2015). The predictive accuracy of such models could thus be improved by incorporating stochastic variation in environmental conditions and allowing for more complex population growth response forms (Botero et al. 2015). We therefore advocate that elevated temperatures and their variability are incorporated into future study design, as these data may be vital to conservation attempts.

## Electronic supplementary material

Below is the link to the electronic supplementary material.

Supplementary material 1 (DOCX 17 kb)
