# Supplementary material for: Response of Development and Body Mass to Daily Temperature Fluctuations: a Study on Tribolium castaneum
Source: Evol Biol. 2016 Feb 24;43:356–67. doi: 10.1007/s11692-016-9375-6 (PMC4960287; doi:10.1007/s11692-016-9375-6)
Supplement: Supplementary file 1 — Supplementary material 1 (DOCX 17 kb) [file 11692_2016_9375_MOESM1_ESM.docx]

**Supplementary Materials**

**Supplementary table 1.** Results of the General Linear Mixed Models analysis for pupal mass of *Tribolium castaneum* in a submodel with time to pupation taken into account as a covariate. Model included dam and sire identities as random effects, unconstrained covariances among experimental treatments, and heterogeneous random effect variances.

| Factor | numerator df | denominator df | Adjusted F | *P* |
| --- | --- | --- | --- | --- |
|  | 1 | 259 | 5582 | <0.001 |
| Temperature | 1 | 2945 | 18.59 | <0.001 |
| Thermal Fluctuations | 1 | 2923 | 1.880 | 0.170 |
| Time to pupation | 15 | 2915 | 7.455 | <0.001 |
| Sex | 1 | 2914 | 22.35 | <0.001 |
| Temperature * Thermal Fluctuations | 1 | 2927 | 5.775 | 0.016 |
| Temperature * Time to pupation | 8 | 2911 | 16.91 | <0.001 |
| Thermal Fluctuations * Time to pupation | 10 | 2923 | 4.984 | <0.001 |
| Time to pupation * Sex | 12 | 2916 | 2.435 | 0.004 |

**Supplementary table 2.** Results of the General Linear Mixed Models analysis for adult body mass of *Tribolium castaneum* in a submodel with time to pupation taken into account as a covariate. Model included dam and sire identities as random effects, unconstrained covariances among experimental treatments, and heterogeneous random effect variances.

| Factor | numerator df | denominator df | Adjusted F | *P* |
| --- | --- | --- | --- | --- |
|  | 1 | 271 | 5846 | <0.001 |
| Temperature | 1 | 2945 | 11.76 | 0.001 |
| Thermal Fluctuations | 1 | 2922 | 3.497 | 0.062 |
| Time to pupation | 15 | 2917 | 6.129 | <0.001 |
| Sex | 1 | 2914 | 20.33 | <0.001 |
| Temperature * Time to pupation | 8 | 2912 | 21.05 | <0.001 |
| Thermal Fluctuations * Time to pupation | 10 | 2922 | 7.708 | <0.001 |
| Time to pupation * Sex | 12 | 2916 | 2.505 | 0.003 |

**Supplementary table 3.** Results of the General Linear Mixed Models analysis for adult body mass of *Tribolium castaneum* in a submodel with Length of pupation taken into account as a covariate. Model included dam and sire identities as random effects, unconstrained covariances among experimental treatments, and heterogeneous random effect variances.

| Factor | numerator df | denominator df | Adjusted F | *P* |
| --- | --- | --- | --- | --- |
|  | 1 | 569 | 3367 | <0.001 |
| Temperature | 1 | 2938 | 18.32 | <0.001 |
| Thermal Fluctuations | 1 | 2913 | 10.46 | 0.001 |
| Sex | 1 | 2909 | 43.87 | <0.001 |
| Length of pupation | 9 | 2928 | 7.791 | <0.001 |
| Temperature * Thermal Fluctuations | 1 | 2921 | 35.06 | <0.001 |
| Temperature * Length of pupation | 3 | 2933 | 7.157 | <0.001 |
| Thermal Fluctuations * Length of pupation | 4 | 2918 | 8.226 | <0.001 |
| Sex * Length of pupation | 4 | 2913 | 2.832 | 0.023 |

**Supplementary table 4.** Results of the General Linear Mixed Models analysis for body mass decrease during the pupation process of *Tribolium castaneum* in a submodel with time to pupation taken into account as a covariate. Model included dam and sire identities as random effects, unconstrained covariances among experimental treatments, and heterogeneous random effect variances.

| Factor | numerator df | denominator df | Adjusted F | *P* |
| --- | --- | --- | --- | --- |
|  | 1 | 743 | 1263 | <0.001 |
| Temperature | 1 | 2944 | 13.74 | <0.000 |
| Thermal Fluctuations | 1 | 2932 | 0.756 | 0.385 |
| Sex | 1 | 2922 | 63.23 | <0.001 |
| Time to pupation | 15 | 2933 | 3.96 | <0.001 |
| Temperature * Thermal Fluctuations | 1 | 2941 | 18.00 | <0.001 |
| Temperature * Time to pupation | 8 | 2926 | 7.066 | <0.001 |
| Thermal Fluctuations * Time to pupation | 10 | 2932 | 4.699 | <0.001 |

**Supplementary table 5.** Results of the General Linear Mixed Models analysis for body mass decrease during the pupation process of *Tribolium castaneum* in a submodel with Length of pupation taken into account as a covariate. Model included dam and sire identities as random effects. unconstrained covariances among experimental treatments and heterogeneous random effect variances.

| Factor | numerator df | denominator df | Adjusted F | *P* |
| --- | --- | --- | --- | --- |
|  | 1 | 1402 | 952 | <0.001 |
| Temperature | 1 | 2940 | 0.145 | 0.703 |
| Thermal Fluctuations | 1 | 2922 | 8.381 | 0.004 |
| Sex | 1 | 2915 | 21.97 | <0.001 |
| Adult | 9 | 2937 | 18.10 | <0.001 |
| Temperature * Thermal Fluctuations | 1 | 2927 | 26.55 | <0.001 |
| Temperature * Sex | 1 | 2923 | 5.728 | 0.017 |
| Temperature * Length of pupation | 3 | 2939 | 44.47 | <0.001 |
| Thermal Fluctuations * Sex | 1 | 2920 | 3.941 | 0.047 |
| Thermal Fluctuations * Length of pupation | 4 | 2926 | 39.15 | <0.001 |
| Sex * Length of pupation | 4 | 2920 | 2.468 | 0.043 |
